# Thoracoscope and thoracotomy in the treatment of thoracic trauma

**DOI:** 10.12669/pjms.35.5.514

**Published:** 2019

**Authors:** Juan Shi, Yucun Wang, Wenzhen Geng

**Affiliations:** 1 Juan Shi, Department of Cardiothoracic Surgery, Binzhou People’s Hospital, Shandong, 256610, China; 2 Yucun Wang, Department of Rheumatology and Immunology, Binzhou People’s Hospital, Shandong, 256610, China; 3 Wenzhen Geng, Department of Cardiovascular Medicine, Binzhou People’s Hospital, Shandong, 256610, China

**Keywords:** Thoracic trauma, Thoracoscopic surgery, Thoracotomy

## Abstract

**Objective:**

To compare clinical effects of thoracoscopic surgery and thoracotomy in the treatment of thoracic trauma.

**Methods:**

Two hundred and fourteen patients with thoracic trauma were randomly divided into a control group and an observation group, 107 in each group. The control group was treated with conventional thoracotomy, while the observation group was treated with thoracoscopic surgery. The operation-related indications, hospitalization, postoperative complications and inflammatory factor level were observed and compared between the two groups. The study was conducted from April 2016 to February 2018.

**Results:**

The duration of operation of the observation group was shorter than that of the control group, the amount of bleeding during operation of the observation group was less than that of the control group, and the postoperative visual analogue score (VAS) of the observation group was lower than that of the control group; the difference were statistically significant (P<0.05). The hospitalization time, time of off-bed activity and time of resuming daily life of the observation group were shorter than those of the control group, and the amount of drainage fluid of the observation group within 24 hours after operation was less than that of the control group; the differences had statistical significance (P<0.05). There was no significant difference in the incidence of postoperative complications between the two groups (P>0.05). The levels of C-reactive protein (CRP), tumor necrosis factor (TNF)-a and interleukin (IL)-6 in both groups after surgery were higher than those before surgery, but the indicators in the observation group were lower than those in the control group (P<0.05).

**Conclusion:**

Thoracoscopic surgery can reduce pains of patients, speed up recovery, and reduce incidence of surgical infection in the treatment of thoracic trauma. It is a safe and effective treatment method, which is worth clinical application.

## INTRODUCTION

Thoracic trauma which is common in clinic is mainly caused by direct violence such as instrument injury, collision injury, falling injury and so on, which may cause different degrees of thoracic trauma. Sites of thoracic trauma are diverse and complex. Severe patients may even suffer from multiple organ injury, having risks of shock and death.^1^,^2^ Once thoracic trauma appears, pulmonary contusion and laceration and intrathoracic hemorrhage may be induced, and most patients may also have symptoms such as fracture, contusion, pulmonary infection, chest deformity and atelectasis, which can aggravate injury.^3^,^4^ A survey has shown that trauma is the leading cause of death among people under 45 years worldwide and chest trauma accounts for 1/4.^5^ In the past, thoracic trauma was usually treated by surgical methods.^6^ However, the traditional thoracotomy is limited in application as it can lead to physical and mental trauma and induce many postoperative complications.^7^,^8^

With the continuous development of thoracoscopic surgery in recent years, its application in the detection and treatment of thoracic injury has become increasingly widespread.^9^ A study has shown that thoracoscopic surgery is superior to traditional thoracotomy in reducing postoperative complications and shortening hospital stay besides advantages of small incision and less pain.^10^ However, the sample size of current studies concerning thoracoscopic treatment for thoracic trauma is small. In addition, thoracoscopy will increase incidence of complications such as pneumonia, atelectasis or iatrogenic diaphragmatic hernia.^11^ Therefore, the effect of thoracoscopy on the treatment of thoracic trauma still needs to be explored. In order to further explore the application value of thoracoscopic surgery, clinical effects of thoracoscopic surgery and thoracotomy in the treatment of thoracic trauma were compared.

## METHODS

Two hundred and fourteen patients with thoracic trauma who were admitted to our hospital from April 2016 and February 2018 were selected. The trauma of the patients was scored using abbreviated injury scale (AIS) and injury severity score (ISS). AIS score no lower than three points or ISS no lower than 16 points was evaluated as severe thoracic trauma. Patients with AIS score no lower than three points and ISS no lower than 16 points, needed surgical treatment, had stable haemodynamics were included. Those who had mild disease condition that could be treated by non-surgical treatment and patients who themselves or whose family members did not wish to participate in the study were excluded. There were 127 males and 87 females, and they aged 19~72 years (average 51.3±8.0 years). They were numbered using random number table and divided into two groups, 107 each. The general data of the two groups had no significant difference, as shown in Table-I. The study was carried out after being approved by the ethics committee of our hospital.

**Table I:** Baseline data between the two groups.

Groups		Observation group	Control group	t/X^2^	P
Sex ratio (male/female)		66/41	61/46	1.083	>0.05
Age (year)		52.15±8.4	50.37± 8.1	0.397	>0.05
ISS		23.66±2.32	24.16±2.08	0.264	>0.05
Time between onset and diagnosis (h)		6.72±2.15	6.81±2.34	0.892	>0.05
Causes for injury [n(%)]	Traffic	56	54	0.843	>0.05
	Fighting	27	28		
	Fall	21	20		
	Accidents at construction site	3	5		

Patients in the control group received traditional thoracotomy. After total intravenous anesthesia, proper surgical incision was made according to the position of injury. Relevant treatment was made after entering into the thoracic cavity, and the specific treatment way was consistent with the observation group. Drainage tube was inserted after surgery, and the incision was sutured. Patients in the observation group received thoracoscopic surgery. Firstly, general anesthesia was performed, and patients were given double-lumen tube (specification: Fr28; Shengguang Medical Products Co., Ltd., China) intubation. The patents kept a lateral position. The 7^th^ intercostal space along the midaxillary line was taken as the surgical approach. The general condition inside the thoracic cavity was probed using the thoracoscope. Blood clot inside the thoracic cavity was cleaned. The injury of the lung was examined. The lung was sutured through incision at the fourth intercostal space under the armpit. Resection was made if the lacerated wound is too severe that could not be repaired. Whether there was other organ injury or bleeding was checked. After repair and hemostasis, the site with rib fracture was fixed under the thoracoscope. The site and number of rib fracture were observed carefully. The rib was pressed under the thoracoscope to find the site of sternal incision suitable for auxiliary fixation. The thoracic walls were cut open layer by layer. The broken ends of fractured bone were exposed under an incision as small as possible. The periosteum of the broken ends was stripped for 2~3 cm, and then anatomical reduction was performed. Damages to the pleura were avoided in the process of operation. Bone lamella was encircled using proper memory ring and covered both ends of the rib after disinfection. The thoracic cavity was washed if the fixation was satisfactory and there was no bleeding. One drainage tube was left.

After surgery, patients in the two groups were sent to intensive care unit and given conventional nursing cares. The changes of vital signs were closely monitored, including pulse, heart rate, consciousness and pupil. Anti-infection, fluid infusion and oxygen nursing were positively given. The treatment and nursing of patients strictly followed aseptic operation principles. Medicine was changed every day. Firstly, the injury site was disinfected and then covered with local transparent adhesive. Patients with local bleeding were in time, and thrombin was used for reducing post-operative traumatic infection. The respiratory function of patients was observed. Blood gas analysis was monitored. Ventilator parameters were adjusted to enhance respiratory tract care to prevent respiratory tract infection. The patients were turned over regularly, and expectoration was paid attention to. After extubation, oxygen inhalation mask was used to ensure that patients breathe smoothly. Nursing of complications was done. Abnormal condition was reported to doctors for timely and effective treatment.

### Observational indicators

Duration of operation and intraoperative bleeding amount were recorded. The postoperative pain of patients was evaluated using visual analogue scale. The hospitalization time, time of off-bed activity, time of daily living recovery, postoperative 24-hour drainage volume and occurrence of complications were recorded. Levels of C-reactive protein (CRP), tumor necrosis factor (TNF)-α and interleukin (IL)-6 were compared before surgery and 24 hour after surgery.

### Statistical Analysis

Data were statistically analyzed using SPSS ver. 20.0. Measurement data were processed by normality test and expressed as Mean±SD if satisfying normal distribution. Data were compared between the two groups using independent sample t-test. Data within the same group before and after treatment was compared using paired t-test. Data which did not satisfy normal distribution was processed by rank sum test. Enumeration data were expressed by frequency; comparison between groups was performed using Chi-square test. P<0.05 indicates that difference had statistical significance.

## RESULTS

The duration of operation of the observation group was shorter than that of the control group, and the intraoperative bleeding amount of the observation group was less than that of the control group, and the postoperative VAS score of the observation group was lower than that of the control group. Differences were statistically significant (P<0.05, Table-II).

**Table II:** Surgical indicators between the two groups.

Groups	Observation group	Control group	t	P
Duration of operation (min)	45.91±23.62	95.63±34.07	13.857	<0.05
Intraoperative bleeding amount (mL)	172.94±134.51	351.42±78.79	19.476	<0.05
VAS score (point)	3.52±1.13	7.19±8.16	6.931	<0.05

The hospitalization time, time of off-bed activity and time of daily living recovery of the observation group were shorter than those of the control group. The postoperative 24-hour drainage volume of the observation group was less than that of the control group. There were significant differences (P<0.05, Table-III).

**Table III:** Hospitalization indicators between the two groups.

Groups	Observation group	Control group	t	P
Hospitalization time (d)	6.14±2.08	8.15±1.26	8.258	<0.05
Time of off-bed activity (d)	1.68±0.24	3.26±1.06	9.531	<0.05
Time of daily living recovery (d)	9.57±1.26	17.27±3.63	17.37	<0.05
Postoperative 24-h drainage volume (mL)	253.22±52.13	280.33±52.30	15.216	<0.05

Levels of IL-6, TNF-α and CRP of both group after operation were higher than those before operation, but the levels of the observation group were significantly lower than those of the control group. Differences had statistical significance (P<0.05, Table-IV).

**Table IV:** Levels of inflammatory factors between the two groups before and after operation.

Groups	Observation group		Control group	

	Before operation	After operation	Before operation	After operation
IL-6 (UG/L)	38.21±8.31	54.24±12.35*^#^	39.11±7.42	82.54±7.41*
TNF-α (UG/L)	17.45±5.31	65.30±14.53*^#^	16.65±3.36	87.56±21.36*
CRP (mg/L)	7.58±1.20	45.36±11.23*^#^	7.65±1.42	67.20±11.27*

* ***Note:***suggests P<0.05 comparing to before operation, # suggests P<0.05 comparing to the control group.

One patient had acute respiratory distress syndrome and one patient had pulmonary infection in the observation group; the incidence of complications was 1.87%. One patient had acute respiratory distress syndrome, one patient had shock and one patient had pulmonary infection in the control group; the incidence of complications was 2.80%. The difference of incidence of complications between the two groups was not statistically significant (P>0.05). All the complications disappeared after symptomatic treatment.

## DISCUSSION

In recent years, the incidence of chest traumatic diseases is increasing year by year. There are many factors leading to chest trauma, including industrial and mining, transportation, natural disasters and so on.^12^ Moreover, chest trauma will seriously affect their respiratory and circulatory functions, lead to a series of pathological changes, and even threaten lives in severe cases.^13^ Surgery is the preferred method for patients with thoracic trauma, most of which are treated by traditional thoracotomy. However, thoracotomy has many disadvantages, such as large trauma, multiple complications and slow recovery, which will not only cause great trauma to the body, but also aggravate pains.^14^ Traditional treatments also include local bandage and assisted thoracic drainage, which can stabilize the injured chest wall and drain the fluid from the chest. However, limited by pains and respiratory movement, patients cannot cough up sputum in the body and may suffer from pulmonary infection and other complications, which may aggravate the disease.^15^ Therefore, it is necessary to find an ideal way to treat chest trauma.

With the continuous development of medical technology, a variety of minimally invasive technologies has developed and has been widely used in clinical practice. Video-assisted thoracoscopy has been recognized by many scholars for its advantages of small trauma and rapid recovery. Thoracoscopic surgery can insert thoracoscope through a new incision or the original closed thoracic drainage opening.^16^ Thoracoscopy can help more intuitively and clearly understand organs such as diaphragm, mediastinum, chest wall and heart and observe whether there is any injury and the actual severity of injury and operation can be implemented according to thoracoscopic results. In terms of operative field, thoracoscopic surgery is better than thoracotomy.^17^ In the traditional thoracotomy, it is usually necessary to make a general judgment on the location of thoracic injury and then determine the surgical incision before surgery. For thoracoscopic surgery, the position of the entrance for thoracoscope is usually fixed, and the condition of thoracic injury will not affect the intraoperative exploration and operation. In addition, incision can be made according to the actual probing result of thoracoscope, which can effectively simplify the operation process and make the whole operation more convenient and fast.^18^ In a study,^19^ 24 patients with posttraumatic hemothorax were treated by thoracoscopic surgery. It was found that thoracoscopic surgery could significantly reduce injuries caused by treatment and the physiological interference caused by operation and it led to a low recurrence rate and few complications. The results of this study also found that patients who received thoracoscopic surgery had shorter operation time and hospitalization time, less intraoperative bleeding amount, lower VAS score and better recovery after operation compared to those who received traditional thoracotomy, which was similar to the results of Xu L et al.^20^ It shows that thoracoscopic surgery can reduce operation time and intraoperative bleeding volume and has a significant promotion effect on the improvement of body functions.

Basic medicine points out that the inflammatory state of body will directly affect operation. If levels of inflammatory factors win the body were too high, it will increase risks of infection and even develop into systemic inflammatory response syndrome which can cause multiple organ dysfunction and is also an important cause of death.^21^ Therefore, in surgical treatment, the over-activation of inflammatory cells should be paid attention to, surgical trauma should be reduced as far as possible, and complications should be reduced. The results of this study showed that levels of IL-6, TNF-α and CRP in the two groups were higher than those before operation, but the levels of these indicators in the observation group were significantly lower than those in the control group. The results were similar to Raghabendran K et al.^22^ It shows that patients who receive thoracoscopic surgery suffered from smaller stimulation, less loss of immune function and a lower stress level, which is beneficial to postoperative rehabilitation.

Although thoracoscopic technique has many advantages, it also requires doctors to have high operational ability and level. Thoracic cavity should be carefully explored during operation, especially mediastinum, posterior sternum and pericardium which are easy to be missed. It is necessary to carefully explore and study the damage of those parts. For some patients with sternum and thoracic vertebra injuries, thorough hemostasis should be carried out in time. For those patients with aortic injuries, CT imaging should be combined, and thoracotomy can be performed if necessary. Alveolar resection should be performed concurrently for patients with bullae.

## CONCLUSION

Thoracoscopic surgery can reduce surgical injuries to patients, reduce the amount of bleeding and the excessive activation of inflammatory cells, and speed up recovery in the treatment of chest trauma. It is a safe and effective method, which is worth clinical application.

### Authors’ Contribution

**JS & YCW:** Study design, data collection and analysis. **JS & WZG:** Manuscript preparation, drafting and revising. **JS:** Review and final approval of manuscript.
